# Cardiopulmonary exercise testing in cardiac rehabilitation: From the reporting form to structured exercise prescription. A proposal from the Italian alliance for cardiovascular rehabilitation and prevention (Itacare-P)

**DOI:** 10.1016/j.ijcrp.2023.200191

**Published:** 2023-06-16

**Authors:** Matteo Ruzzolini, Marco Ambrosetti

**Affiliations:** a Cardiology Division, Fatebenefratelli-Isola Tiberina Hospital, Rome, Italy; b Cardiovascular Rehabilitation Unit, ASST Crema, Santa Marta Hospital, Rivolta D'Adda, Italy

**Keywords:** Cardiopulmonary exercise test, Exercise prescription, Cardiac rehabilitation

## Abstract

The cardiopulmonary exercise test (CPET) is the gold standard for the diagnostic evaluation of exercise intolerance, as for individualized prescription of structured physical training. Exercise is a core component of cardiovascular prevention and rehabilitation activites, but unfortunately the limited availability of CPET-derived informations often leads to unpowered program's prescription in real life. The Italian Alliance for Cardiovascular Rehabilitation and Prevention (ITACARE-P) has developed a CPET reporting form specifically oriented to exercise prescription, in order to facilitate interventions on lifestyle and during phase II/phase III cardiac rehabilitation programmes. The ITACARE-P CPET reporting form includes a limited number of key variables for clinical practice and individual domains of exercise intensity, suitable both for threshold-based and range-based aerobic training protocols. The adoption of the ITACARE-P CPET reporting form could improve non-pharmacological intervention in preventive cardioloy and facilitate collaborative research on physical training within the network of cardiac rehabilitation facilities.

## 1 Introduction: why this proposal?

Structured physical training represents a fundamental core component of Cardiac Rehabilitation (CR) [1] and of cardiovascular prevention activities aimed at healthy subjects, carriers of risk factors or known cardiovascular disease patients [2,3]. Cardiopulmonary exercise testing (CPET) has a gold standard role as a method for assessing global functional capacity, identifying limitations in exercise tolerance, and setting parameters for aerobic training [4].

Although adequate experience and autonomy in performing CPET and planning a personalized physical training program must be part of the core curriculum of each clinical cardiologist [5], particularly of the cardiologist dedicated to cardiovascular prevention [6], unfortunately in routine practice referral and execution rates of such testing are low. As a consequence, the prescription of training protocols in cardiovascular patients is frequently based on suboptimal parameters, mostly obtained from the classic ergometric test or rating of perceived exertion (RPE) scales [7].

The reasons for this underuse - with consequent depowering of the prevention/rehabilitation program in terms of risk profile reduction, disability restraint and prognostic improvement - are various and may be linked to a lack of familiarity with the CPET and the large amount of information obtainable with this test. The need for an easy-to-use interpretative approach based on simple algorithms have already been advocated, leading to recommendations with particular reference to diagnosis and prognostic prediction in the heart failure setting [8].

Inside of CR programs, however, there is a need for a CPET report model which, in addition to limited but exhaustive set of variables useful for interpreting the global functional capacity and specific exertional limitations (cardiac, ventilatory, pulmonary, muscular), gives also back personal intensity domains of aerobic training. In fact, the intensity of aerobic effort represents one of the main determinants of a personalized training program, thus influencing expected benefits in terms of performance, quality of life, and prognostic impact. Therefore, in our opinion, it's crucial to use an appropriately reported CPET to prescribe a training program embracing individual response to exercise, physical and clinical characteristics of the subject, his possible previous sporting history, as well as current therapy.

Not by chance, main decision support systems that have been developed to assist the cardiologist in preparing a personalized training program - such as the EXPERT tool of the European Association of Preventive Cardiology (EAPC) [9] – often require the input of parameters obtainable only from the CPET, such as the oxygen consumption value at maximal effort and its percentage with respect to the predicted.

For these reasons, the Italian Alliance for Cardiovascular Rehabilitation and Prevention (ITACARE-P) carried out a working group aimed at developing a shared report model between cardiologists and exercise experts, proposed as a tool to be used in daily practice. This model adopts classic theoretical bases of implementation and interpretation of the CPET (whose explanation in detail goes beyond the scope of this document and can be found in the attached bibliography [10,11]), while simplifying the amount of data provided from this test, and focusing attention on the most relevant aspects for the prescription of structured training. In this perspective, several modern variables such as the oxygen uptake efficiency slope (OUES), the circulatory/ventilator power, and the VO2 recovery kinetics – despite their growing utilization – were not included in the model, to facilitate “newcomer users” of CPET in CR.

## 2 Who can use the CPET reporting form

This template was primarily designed to guide structured CR programs, both in the phase immediately after an acute event (if the patient's conditions allow it), and in the chronic phase, as well as for conducting maintenance programs for long-term results. In this contest, it represents an essential tool for the physician that prescribes physical training and the physiotherapist, in order to perform an accurate functional evaluation, administer exercise, measure the outcome of the intervention and prepare a suitable program coupled with educational counselling.

The identification of training intensities accompanying the report is not only useful for conducting the classic center-based CR program but, properly validated, can also support the general practitioner, the sports clinicians or other acknowledged contexts (e.g. “health's gyms” or similar) for the promotion and management of structured training in the long term, as well as promoting the self-management of the patient, especially in the case of highly motivated subjects or recreational athletes. Finally, the model is also potentially suitable for theoretical and practice teaching activities, in particular as regards the pathophysiology of reduced exercise tolerance and the professional prescription of physical training, which can be used in updating and training process of physicians, physiotherapists, and health operators dealing with therapeutic physical exercise.

## 3 How to use the CPET reporting form

The reporting form is released by ITACARE-P without constraints of use, in text form that can be further adapted and integrated locally in hospital informatic systems, even with automatic generation of some outputs (for example, the individual table of intensity domains). ITACARE-P hopes for a widespread use of the report model within the cardiovascular rehabilitation community, also promoting comparison initiatives on the feasibility and practical value of this report, useful to multi-center collaborative research initiatives.

## 4 Structure of the report template

**Fig. 1:**
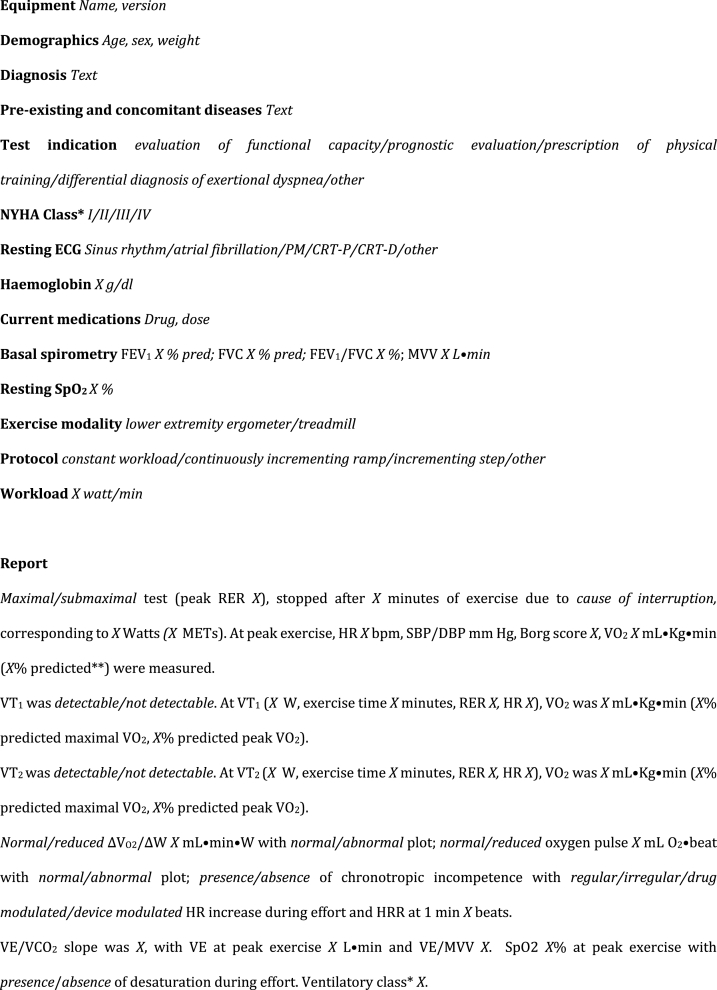

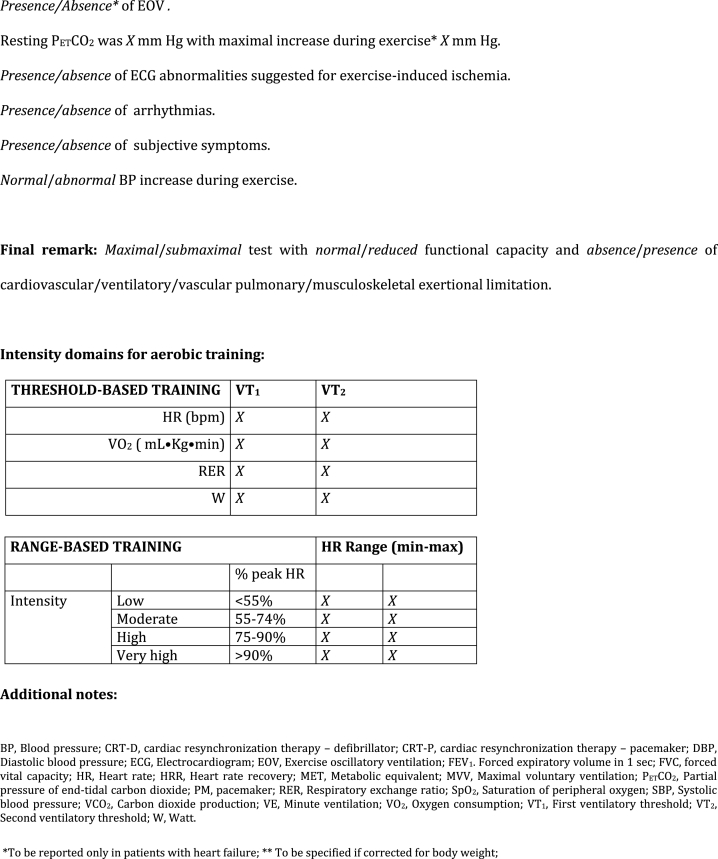
ITACARE-P reporting form of cardiopulmonary exercise testing for training prescription.

The ITACARE-P report template (Fig. 1) comprises the following sections.

1) Description of technical instruments, test indication, anthropometric measurements, clinical variables, and current therapies
2) Basic spirometry data
3) Testing protocol (modality of exercise, selection of total workload and work rate increase)
4) Systematic reporting and final remarks
5) Individual identification of intensity domains for aerobic training based on ventilatory thresholds (*threshold-based training*) and percentage of peak HR (*range-based training*).

In view of the large number of CPET variables, the reduction to a few key variables enables all exercise precribers 1) to determine whether and to what extent there is impaired functional capacity, 2) to evaluate cardiovascular, ventilatory, pulmonary vascular or musculoskeletal limitations, and 3) to identify individual domains of exertional intensity. Certain variables are condition specific, as the case of ventilatory class or oscillatory pattern in heart failure patients. Unless otherwise specified, variables warrant assessment in all conditions. For the sake of simplicity, the ITACARE-P report template does not provide emerging CPET variables such as the oxygen uptake efficiency slope (OUES), the circulatory power, and VO2 onset and recovery kinetics. For the same reason and to avoid ambiguity in data interpretation, the report template simply adopts the terms of “first” (VT_1_) and “second” (VT_2_) ventilatory thresholds, with no other definitions such as “anaerobic threshold”, “lactate point”, “respiratory compensation point”, or “critical power”. VT_1_ and VT_2_ mark the limit between moderate and high intensity domains and are now considered as the preferred drivers for exercise prescription. However, the template maintains also the possibility to have parameters for range-based training protocols, derived from the 2020 ES C guidelines of on sports cardiology and exercise in patients with cardiovascular disease [3], suitable for those cases in which ventilatory theresholds could not be detected for any reason. Whatever the adoption of threshold-based or range-based exercise protocols, usual adaptations are needed when adopting treadmill exercise (due to difficulties on load prescription in Watts), or when translating data from incremental testing to endurance continuous exercise. Similar precautions should be applied when using heart rate recovery data in challenging patient groups, such as those with atrial fibrillation or heart transplantation.

Concerning oxygen consumption at VT_1_ and VT_2_, both per cent-predicted maximal VO_2_ and per cent peak VO_2_ are requested, since potentially exploring two different domains (namely prognosis and patient deconditioning respectively).

## 5 Conclusion

Among common indications for CPET, guiding individual exercise training represents one of the most useful application for cardiovascular prevention and rehabilitation activities. The ITACARE-P reporting form provides an easy-to-follow approach to translate CPET data into an individual program for aerobic exercise training in routine practice. By appropriate local adaptation, it could improve patient management and enhance familiarity and application of this goal standard testing modality.

## Credit author statement

Marco Ambrosetti: Conceptualization, Methodology, Matteo Ruzzolini: Writing- Original draft preparation, Writing- Reviewing and Editing.
